# An explorative qualitative study on acceptability of physical activity assessment instruments among primary care professionals in southern Sydney

**DOI:** 10.1186/s12875-016-0536-6

**Published:** 2016-09-22

**Authors:** Shona Nicole Dutton, Sarah May Dennis, Nicholas Zwar, Mark Fort Harris

**Affiliations:** 1 Centre for Primary Health Care & Equity, University of NSW, Level 3, AGSM Building, Sydney, 2052 Australia; 2 Faculty of Health Sciences, University of Sydney, Room: O206, O Block, 75 East Street, Lidcombe, NSW 2141 Australia; 3 School of Public Health and Community Medicine, University of NSW, Level 3, Samuels Building, Sydney, 2052 Australia

**Keywords:** Acceptability, Family practice, General practitioner, Practice nurse, Physical activity, Physical activity assessment, Questionnaire

## Abstract

**Background:**

There are a substantial number of instruments for primary-care clinicians to assess physical-activity (PA). However, there are few studies that have explored the views of clinicians regarding comparative acceptability and ease of use. A better understanding of how clinicians perceive instruments could help overcome barriers, and inform future interventions. This study explored the acceptability of five PA-assessment instruments amongst a sample of Australian primary-care clinicians, including family-physicians (FP) and practice-nurses (PN).

**Methods:**

A purposive sample of FPs (*N* = 9) and PNs (*N* = 10) from eight family-practices in southern Sydney consented to participate. Stage-1 involved semi-structured interviews with participants to select preferred instruments. An analysis of the two preferred instruments was conducted as Stage-2, to identify differences in instrument purpose and content. Stage-3 involved participants using the two instruments, selected from Stage-1, for 12-weeks. At the end of this period, semi-structured interviews were repeated to explore clinician experience.

**Results:**

Clinicians indicated preferences for the GP-Physical-Activity-Questionnaire and 3-Questionnaire Physical-Activity-Questionnaire. These instruments demonstrated distinct variations in content, theoretical orientation, and outcome measures. Reasons for preference included; variations in individual clinician PA levels, knowledge in PA-assessment and instrument features.

**Conclusion:**

Findings demonstrated two instruments as preferred. Reasons for preference related to internal characteristics of clinicians such as variations in the level of individual PA and external circumstances, such as instrument features.

## Background

Evidence-based guidelines have been developed to support Australian primary care clinicians to address physical activity (PA) behaviour change in their patients [1, 2]. Despite evidence demonstrating the importance of implementing brief interventions, uptake is less than satisfactory with as few as 30 % of primary care encounters involving PA assessment [3, 4]. These data highlight the need for routine and consistent assessment of PA within clinical settings to improve identification of insufficient PA, and instigate behavior change. Understandably, there are many challenges to routine PA assessment within clinical settings and subsequently, a range of tools have been developed. Physical activity questionnaires are used to determine PA status, by providing self-report responses to questions regarding a selection of PA domains [5, 6]. Despite some evidence indicating limitations of self-report, they remain the most cost effective and pragmatic option for assessing patient PA behaviour, within primary care settings [7–9]. However, research indicates a degree of analytical rigour when using self-report PA assessment instruments, [5, 6, 10, 11]. Evidence has demonstrating strong correlations and agreement with other construct criteria measures for vigorous-intensity PA. Discriminant validation studies have also shown that questionnaires have usefully classified patients in rank order according to activity level [5, 6, 10, 11]. This reinforces the value of PA assessment instruments in primary care settings, specifically for risk factor identification and behaviour change interventions.

In Australia, a range of policy initiatives have led public health approaches to reduce the prevalence of physical inactivity. These include the introduction of inaugural PA guidelines (1995), which were updated in 2015, and introduction of national surveillance activities [12]. Inter-government and inter-sectoral approaches have been implemented through the Active Australia and Strategic Inter-Government forum on PA and Health (SIGPAH) [13]. More recently, the Australian Government committed $932 million between 2009 and 2018, for strategies to prevent disease through the National Partnership Agreement for Preventive Health (NPAPH) [14–16].This work will encourage the adoption of healthy behaviours, including PA [14–16]. The Royal Australian College of General Practitioners (RACGP) responded to the need for PA policy in the primary care setting by establishing guidelines for prevention of chronic disease [17] and, guidelines for preventive activities in general practice, Both resources have been designed to support primary care clinicians to implement preventive activities [17, 18].

There are several barriers identified as limiting the uptake of preventive activities, including PA assessment within primary care settings [8, 19, 20]. In response, researchers have focused on ways to support clinicians to apply the National PA Guidelines through interventions assessing patient PA [9, 21, 22]. Since the introduction of the Australian PA Guidelines in 1999 [23], a number of PA assessment questionnaires have been developed for use in primary care [22, 24–27]. However uptake has been suboptimal with evidence indicating a number of barriers experienced by clinicians including; time constraints; knowledge about PA; inadequate skills with interpretation of PA assessment; and capacity limitations of the practice [24, 28–30].

Other than identification of general barriers to uptake of PA assessment, little is known about the acceptability of these instruments for meeting the needs of the Australian primary care setting. One study by Smith et.al [24] determined the validity and reliability of the 2Q and 3Q instruments in an empirical design; but did not determine uptake in routine practice. Further research has focused on the population wide monitoring and reporting of PA, rather than application in primary care, whilst others have not been investigated in an Australian context [25, 31, 32].

Identifying interventions that help primary care clinicians to conduct PA assessment, whilst taking into consideration limitations on their capacity, has been identified as a key success factor in the uptake of guidelines [33]. To date, researchers have placed emphasis on overcoming limitations in family physicians (FP) time such as providing new instruments that are briefer in length and content [24]. Auxiliary approaches have included providing questionnaires in alternative formats such as electronic templates which are compatible with medical software and linking the assessment to (clinician) incentive funding such as Medicare Health Assessments and care plans [34]. However, there has been little noteworthy change in the uptake of PA assessment in family practice [24, 28].

This study sought a better understanding of how clinicians perceive assessment instruments and how these were influenced by clinician factors and their experience using the instruments in practice in order to inform future PA interventions.

### Aims

This study aimed to determine the following;

- Identify instruments preferred by family practice clinicians, to administer amongst patients in routine practice.
- Ascertain reasons for clinician preferences before and after using the instrument.
- Identify intrinsic and extrinsic variables that influence clinician uptake of physical assessment amongst patients.

## Methods

A purposive sample of FPs (*n* = 9) and practice nurses (PNs) (*n* = 10) from eight family practices from one primary care organization (PCO) in southern Sydney were identified. FPs had referred a patient to the PCO’s GP Exercise Referral Scheme (GPERS) in the previous six months were eligible to participate in the study. PNs from practices with a FP, who had previously referred to the GPERS program in the six months prior to the study, were also eligible to participate. The GPERS Scheme was a local initiative where FPs could refer physically inactive patients for PA assessment and exercise prescription, with an exercise physiologist. Although PNs were not eligible to directly refer to the GPERS Scheme, they were included with the scope of this study because of their potential role in lifestyle risk factor management within the primary care setting. Of the 214 FPs and 46 PNs practicing in the region, 123 FPs and 32 nurses were eligible to participate.

FPs and PNs were sent an invitation letter and information sheet explaining the purpose of the study. This was followed up with a visit from the investigator (SND) who explained the project in detail and obtained their written, informed consent.

The study was conducted in three stages. Stage-1 involved semi-structured interviews to identify two PA assessment instruments, preferred by the clinicians. Stage-2 involved conducting a detailed analysis of the two preferred instruments (from stage 1) relative to the features of each instrument. Stage- 3 was the implementation of the two preferred instruments over a period of 12-weeks. At the end of the implementation period, semi-structured interviews were conducted to explore clinician experience and identify the two highest ranked preferences.

### Stage-1

The FPs (*n* = 9) and PNs (*n* = 10) took part in semi-structured interviews with the investigator (SND). Demographic data were collected for each participant including; age, gender, profession and practice location. Clinician PA behaviour was assessed by the chief investigator; a tertiary trained Exercise Physiologist who determined the frequency and intensity of PA undertaken, over the previous or usual week. The responses provided by clinicians were used to determine whether they were sufficiently physically active, against the Australian National PA Guidelines [23].

Following collection of demographic data, participants were provided with copies of five commonly used PA questionnaires to review, and were asked a series of questions about their preferences. The following five instruments were selected based on their potential for use in Australian family-practice;

- Active Australia (AA) [35]
- Occupational Sitting and PA Questionnaire (OSPAQ) [25]
- 2-Question PA Questionnaire (2Q) [24]
- 3-Question PA Questionnaire (3Q) [24]
- General-practice PA Questionnaire (GPPAQ) [32]

This study aimed to determine clinician preferences for a range of PA assessment instruments. It called for clinicians to draw on insight into their patient population, and practice systems to determine which instrument would be the best fit for their individual situation. Clinicians were considered as having experience in assessing patient PA behaviour, determined by previous referrals to the GPERS program. Former methods used to assess PA, or the frequency at which this occurred was not determined because of potential recall bias. Clinician knowledge of PA assessment was determined by their PA status. The process for determining PA status is outlined in the methods for Stage-1 of this study.

The interviews were guided by a schedule (Appendix) of open-ended questions to explore the participants’;

- Instrument preferences,
- Understanding and confidence in PA assessment, and
- Perceptions of barriers to assessing PA.

### Stage-2

An analysis of the two preferred instruments identified from Stage-1 was conducted to identify differences in instrument purpose and content. Variables considered in this analysis included; theoretical orientation, length of the instrument including the number of questions and estimated time taken to complete, scoring or outcome measures, terminology and/or language used within the content of the instrument, types of PA considered (e.g. planned, incidental, work and leisure) and the use of explanatory text such as examples and scenarios.

### Stage-3

The two instruments ranked highest from Stage-1 were implemented by clinicians in routine practice, over a 12-week period. At the end of the 12-week period, there was a second round of semi-structured interviews to determine participants’ satisfaction and experiences of using the selected instruments. There was one FP and one PN that were unavailable to participate in the follow-up interviews leaving eight FPs and nine PNs who took part. The interviews were guided by a schedule (Appendix) and the questions covered;

- Preferences between the two (selected) instruments.
- Understanding and confidence in PA assessment using the two (selected) instruments.
- Exploration of their perceptions of barriers to assessing PA using the selected instruments.

All interviews were conducted in 2011 and were audio recorded and field notes made. The interviews were transcribed verbatim. Ethical approval was granted by the University of New South Wales Human Research Ethics Committee (HREC 11068).

### Analysis

Content analysis was conducted following the framework analysis approach [36]. FP and PN interviews were analysed together. SND read and re-read all transcripts and coded emergent themes and subthemes using the 18 theoretical domains and 112 constructs from the Theoretical-Domains-Framework (TDF) [24, 37–39]. The TDF was selected because of its capacity to integrate 33 constructs, across 18 domains of behavioural determinants, covering the full range of current scientific explanations for human behaviour (i.e., ‘Knowledge’, ‘Skills’ and ‘Social/professional role and identity’). The coding was discussed with members of the research team and modified following discussions.

To ensure analytical rigour, a second iteration of this process was performed, with re-review of transcripts to identify any important quotes or subthemes missed or misallocated. It was noted whether subthemes arose solely by FPs, PNs or both. The final synthesis and interpretation involved considering each theme/domain and subtheme, in the context of the whole set of interviews. The strongest domains were those mentioned by most practitioners; were discussed at greatest length; and/or judged by the investigators to be invested with considerable intensity, passion, or sentiment by clinicians.

## Results

### Health Professional characteristics

A total of nine FPs and ten PNs took part in the interviews in Stage-1 and eight FPs and nine PNs in Stage-3. There was one FP and one PN that were unavailable to participate in follow-up interviews, due to leave. Participants represented eight group practices and with an equal proportion of small (four or less FPs) and large (five or more FPs) practices. The characteristics of the participants are detailed in Table 1.

**Table 1:** Clinician characteristics

Characteristic	FP (*n* = 9)	PN (*n* = 10)
% female	45 %	100 %
% working in small (≤4 FPs) practice	4	6
Practice size – Large (≥5 FPs)	5	4
% physically active *(i.e. meets PA guidelines)*	100 %	40 %

Clinicians were classified as either meeting or not meeting the Australian PA Guidelines of 30-minutes or more moderate intensity PA on most days of the week. A total of 68.4 % (13/19) of clinicians indicated that they were currently physical active, 100 % of males and 57.1 % (8/14) of females.

### Stage-1 – Questionnaire Preferences

The majority of clinicians (88 % FPs and 100 % PNs) interviewed in Stage-1 preferred the GPPAQ [32]. A ranking process determined the GPPAQ and 3Q [24] as most preferred, from the original selection of five instruments and they were used in the second stage (Table 2).

**Table 2:** Questionnaire preferences for clinicians at Phase 1 and Phase 2 of semi-structured interviews.

	FP			PN		
	Phase 1		Phase 2	Phase 1		Phase 2
	1st Preference (*n* = 9)	2nd Preference (*n* = 2)	Aggregate preference (*n* = 8)	1st Preference (*n* = 10)	2nd Preference (*n* = 4)	Aggregate preference (*n* = 9)
AA	0	1		0	0	
OSPAQ	0	0		0	0	
2Q	1	0		0	0	
3Q	0	1	4	0	4	1
GPPAQ	8	0	4	10	0	8

### Stage 2 – Preferred instrument analysis

The instruments selected in Stage-1 were (1) GPPAQ [40] and (2) 3Q [24] and were different across a range of variables. The GPPAQ was longer in length than the 3Q, and used explicit examples of incidental and planned PA. This included specific reference to PA undertaken in an occupational setting. Whereas the 3Q was briefer in length, it contained technical terminology, typically used by exercise professionals. A comparison of selected variables for the two preferred instruments is provided in Table 3.

**Table 3:** Preferred instrument analysis, across selected variables.

	Preference 1: GPPAQ	Preference 2: 3Q
Theoretical orientation	▪ Validated instrument designed to produce a short measure of PA in primary care patients aged 16–74 years. ▪ Administration of the instrument: ◦ FP ◦ PN ◦ Patient ◦ Other health care professionals [32]	▪ Designed for epidemiological surveillance purposes and adapted for use in family-practice. ▪ Administration of the instrument: ◦ FP ◦ PN ◦ Other health care professionals [18]
Length (number of questions)	▪ 7 questions. ▪ Additional sub-questions. ▪ Estimated completion time between ≤1 minute [32].	▪ 3 questions. ▪ Estimated completion time between ≤1 minute.
Outcome measures	▪ Provides a simple, 4 level PA index (PAI); Inactive, Moderately Inactive, Moderately Active or Active.	▪ Assigns patients based on outcome score to one of four categories; Minimal, Low, Adequate or High.
Terminology and/or language	▪ Simple language. ▪ Terminology typically used amongst lay-people.	▪ Technical used by exercise professionals. ▪ Terms used obtain unique definitions specific to PA assessment e.g. Vigorous and Moderate Intensity.
Range of PA settings considered	▪ 5 Occupational settings. ▪ 3 Planned exercise settings. ▪ 2 Home-based incidental settings.	▪ Discrete suggestions of incidental and planned exercise. ▪ No reference to specific environments or situations.
Use of explanatory text such as examples and scenarios	▪ 28 explicit examples, within scenarios.	• 9 single-term examples of types of exercise e.g. jogging, walking or digging. • Discrete definition for vigorous and moderate activity.
Use of explanatory text such as examples and scenarios	▪ 28 explicit examples within scenarios	• 9 single-term examples of types of exercise Discrete definition for vigorous/moderate PA

### Stage 3 – Questionnaire Preferences

After implementing the instruments in Stage-3, preferences changed amongst some clinicians, particularly amongst those clinicians (FP and PNs) who were more physically active. In Stage-1, 89 % (*n* = 9) FPs preferred the GPPAQ instrument. In Stage-3 this proportion changed (for FPs) to an even preference for GPPAQ and 3Q.

### Key themes derived from the Theoretical Domains Framework

Not all domains from the TDF were found to be relevant to the context of the interviews. Relevant domains and themes were grouped into (1) Intrinsic or (2) Extrinsic variables. Intrinsic variables are those inherent to the clinician. Extrinsic are fundamentally, external influencers. Data has been presented according to the themes identified from the TDF below;

## Intrinsic variables

### TDF Domain: Knowledge

In the context of this study, the domain “knowledge’ refers to clinician knowledge and perceived competency about PA assessment/advice [37]. Clinician feedback demonstrated a link between the following three themes;

- Theme 1: Clinician knowledge/competency
- Theme 2: Clinician individual characteristics
- Theme 3: Instrument design/content

#### Theme 1: Clinician knowledge/competency

Clinician knowledge and understanding of PA was determined based on their current PA status, and their awareness of Australian PA guidelines, including their understanding of terminology associated with PA assessment e.g. differentiating between vigorous and moderate PA. A participating PN who was considered as having less knowledge and/or understanding of PA domains indicated that the 2Q and 3Q instruments were limited in the information they provided, whereas the GPPAQ provided more detail to conduct the assessment *“…there’s just not enough information in there and these are a bit more detailed …” *(PN6). An FP, also considered to be less knowledgeable of PA suggested that the same instruments [2Q and 3Q] *“…took more concentration to work out; I had to go back over the questions…” *(FP5)

The analysis of the two preferred instruments carried out during Stage-2 of the study found the GPPAQ to offer rudimentary support for clinicians less knowledgeable of PA, whereas the 3Q instrument suited those more familiar with the mechanisms of PA assessment.

Clinician knowledge/competency regarding PA appeared to influence their preference for instruments. Clinicians with less knowledge about PA preferences were more likely associated with the GPPAQ, the reverse was the case for 3Q. For example, several clinicians highlighted that the GPPAQ instrument provided terminology or wording that was “… *more specific with asking exactly what exercise” and comments that the GPPAQ instrument “… was more specific*.” (PN5).

#### Theme 2: Clinician individual characteristics

Clinicians meeting national PA guidelines showed greater understanding of PA, and had a preference for the 3Q rather than the GPPAQ, in Stage-3, whereas those less physically active preferred the GPPAQ, linked to its ability to guide the assessment process.

#### Theme 3: Instrument design/content

Participant responses provided insight into the knowledge and confidence of clinicians, regarding PA assessment. This was closely linked with the design, and content of instruments. The GPPAQ featured elementary style language, typically used in lay language. It was longer in length and used explicit examples for incidental and planned PA including reference to occupational activity (see Table 3).

Participant’s referred to how their preferred instrument supported inadequacies, or limitations faced in conducting PA assessments. Specifically, PNs referred to the absence of technical terminology such as “vigorous” and “moderate” intensity in the GPPAQ. Several clinicians highlighted that the GPPAQ provided terminology that was “… *more specific with asking exactly what exercise*” and comments that the GPPAQ *“… was more specific*.” (PN5)

Just over half of all clinicians reported using the instrument as a prompt/guide during the assessment, indicating that the “… [GPPAQ] *examples helped explain what was meant*” and were “… *clearly written [with]… good examples of what they would expect each types of activity to include*.” (FP5)

The GPPAQ content used limited technical (PA) terminology and used examples for the subject to consider, such as work, leisure and planned PA. For example, one clinician said that the GPPAQ ‘…*gets people to give a bit of a depiction of how their work is and exactly how intense their work is … it also breaks down the PA outside of work fairly accurately too…. somewhat easier for the patient to interpret than some of the other ones… [gives]…more of an idea of what they’re actually doing rather than them just saying I do regular exercise*.” (FP4)

Comments regarding the content and design of each instrument included “…*examples helped explain what was meant*” (FP5) and were “… *clearly written [with]… good examples of what they would expect each types of activity to include*.” (PN4). In addition, the scope of the instruments and types of patients that were considered also influenced preferences. An important distinction made in relation the GPPAQ included the assertion that the “… *GPPAQ was broader based, so it covers the employment side of things as well as the things that you do for leisure as opposed to the other one seems to be more just what you do for leisure, really*.” (PN4).

### TDF Domain: Beliefs about capabilities

The intrinsic beliefs and capabilities of clinicians, about their ability to execute PA assessment was linked to instrument preferences. There were two themes associated with this domain;

- Theme 1: Ability to motivate patients
- Theme 2: Confidence and familiarity

#### Theme 1: Ability to motivate patients

There was reference regarding clinician’s ability to motivate the patient for successful behaviour change and the role the instrument played in supporting this. Some clinicians thought the instruments “…*helped motivate these patients to exercise if they weren’t already*”. The questionnaires prompted patients to think about their activity. A participating PN recalled a patient saying “*You know, I think I should be doing more, I should be doing more*”. (PN5)

#### Theme 2: Confidence and familiarity

Clinicians discussed how they would link the use of the instrument to existing procedures or activities within their practice. They reflected on current processes/systems in place, and how the questionnaire would fit within this framework so that it could conform to existing processes.

One of the FPs referred to the similarities between the 3Q instrument and current practice. She highlighted that “*This is similar to the way I’m already approaching patients… I suppose I’m biased because it’s something that I’m familiar with and that’s the way I do it, um and it can lead on to some advice I guess*…” (FP6)

## Extrinsic variables

### TDF Domain: Social/professional and role and identity

The analysis indicated that clinicians maintained a professional responsibility to facilitate PA assessment. Professional training, knowledge and competencies provided clinical knowledge of the benefits associated with PA. There were two themes that emerged from clinician feedback that related to this domain;

- Theme 1: Patient selection
- Theme 2: Leveraging external factors during consultations

#### Theme 1: Patient selection

Extrinsic variables included the professional responsibilities of the clinicians and how the instrument supported this role. Clinicians referred to patients within a strata or a demographic classification e.g. patients with established chronic conditions, gender, age or social mediums, such as unemployed, mothers and elderly.

There were differences between clinician roles and responsibilities and how they referred to implementing their preferred instrument. Both FPs and PNs indicated that PA assessment was undertaken with select patients, however for FPs, selection was undertaken on an incidental basis rather than pre-emptive planning. That is, as patients presented for consultations, the clinicians elected to conduct an assessment if they felt there was a specific clinical need. In essence, this stratified patients, albeit incidentally for assessment rather than assessing the practice population, in an all-encompassing approach. One FP, mentioned that *“…whenever I go through blood test results and there’s something that’s a little bit abnormal… high cholesterol, borderline sugar, it usually does prompt a discussion on exercise….middle aged patients who are slightly overweight…”* (FP4)

Dissimilar to FPs, PNs used the preferred instrument(s) within formal practice-based initiatives such as health assessments. This was evident when discussing the type of consultations or patients they would likely initiate PA assessment. A nurse whose primary role was to conduct 75 year old health assessments for her practice selected the GPPAQ instrument because it “… *would incorporate the retired people*” (PN3). Another nurse mentioned that her role focused on women’s health. This nurse preferred the GPPAQ instrument because “*It covers traditional women’s activities like housework better than Questionnaire 3* [3Q]…” (PN2)

#### Theme 2: Leveraging external factors during consultations

The use of specific situations where the clinician could introduce or initiate PA assessment, were highlighted during interviews. Clinicians referred to the use of the preferred questionnaire(s) during consultations where they could initiate a discussion about PA under the guise of something else such as health assessments, poor pathology results, diabetes cycle of care activities and care planning. One FP commented that “… *whenever you go through blood test results there’s often something that’s a little bit abnormal, you know high cholesterol, boarder line sugar, it usually does prompt a discussion on exercise. … it would be very useful for that situation*.” (FP4)

### TDF Domain: Innovation

Innovation referred to the use of the PA assessment instrument as a tool to discourage/encourage the development of PA assessment skills or behaviour. There were two themes that emerged from the data that related to this domain;

- Theme 1: Support tool for conducting/initiating PA assessment
- Theme 2: Adaptive behaviour to support improved competency

#### Theme 1: Support tool for conducting/initiating PA assessment

Clinicians referred to using the instrument as a mechanism for starting a conversation with the patient about PA, rather than raising with topic independently. In one case, the GPPAQ was used as “… *a springboard …It kind of led on to other things*.” (PN10). In addition, there was reference to the questionnaires acting as a prompt during their consultation with patients, initiating thought about activity. A participating PN referred to a consultation with a patient where PA behaviour was discussed. The PN recalled that the conducting the PA assessment using the GPPAQ enabled patients to independently realise they were insufficiently physically active. This PN recalled patient responses “…y*ou know I think I should be doing more, I should be doing more*” that kind of think came up. (PN10)

#### Theme 2: Adaptive behaviour to support improved competency

After using the instruments in Stage-3, preferences changed amongst some clinicians from the GPPAQ to the 3Q. This was particularly evident amongst clinicians with higher knowledge/perceived confidence of PA assessment. This indicated a period of adaptation and heightened understanding of the concepts of PA assessment. Supporting the premise that a degree of adaptation occurred between the two study points (Stage-1 and 3), amplifying clinician competency. For example, a clinician referred to their interaction with a patient during the assessment and how they “… *found the 3Q one a little harder to understand at first, but we just read it through a few times and then it was no problem*.” Another clinician referred to the use of examples in the GPPAQ “…*were good, because that way they [the patients] realised what vigorous was*. ” (PN4)

### TDF Domain: Innovation strategy

Innovation strategy refers to how the PA instruments encouraged or discouraged the execution of PA assessment for each clinician. Clinicians indicated that the brevity of the instrument was not indicative of the time taken to complete the questionnaire, and inconsequential in deciding their preferences. Whilst time was raised as a consideration, it was associated with how quickly or efficiently they could complete it the assessment. This was linked to clinician knowledge and confidence and how this would impact the time taken to complete an assessment. This was linked to their ability to their understanding of the content of each instrument. Almost half (47.4 %, 9/19) of all clinicians referred to the support the instrument(s) provided them using phases such as “…it took a little bit longer but I’d still prefer this one [GPPAQ].” (PN6) and “… I’d rather do [GPPAQ] and get that much more info ” (FP2).

### TDF Domain: Social influences

In the context of this study, social influences referred to interpersonal variables that influenced clinician knowledge/competency of PA assessment.

Analysis identified associations between clinicians who were physically-active and their preference for the 3Q instrument. This was particularly evident in Stage-3, when clinicians had used both instruments for the period of the intervention (12-weeks). These differences indicated a variation in the competency of clinicians administering the instruments. Administration of the 3Q necessitated a proficiency in PA assessment variables. The link between preference and PA status possibly relates to prior knowledge, perceived confidence, and/or personal experience with PA.

The study did not determine the number of PA assessments undertaken by clinicians during the intervention period. The primary purpose of the study was to determine clinician preferences following a period of ‘testing’ the preferred instruments (between Stages 1 and 3). This process was to validate initial preferences stated in Stage-1 interviews. Initially, the frequency of PA assessments undertaken during the testing phase was not considered when designing the study. Some clinicians changed their preferences between Stage 1 and Stage 3. This is indicative that a period of adaption or learning occurred after using the instruments, following their initial impressions of each instruments. This study did not focus on variations in exposure to each instrument. Further research is required to investigate the educational requirements or variations that occur to increase clinician knowledge on this topic. This has been added to the further research section in the conclusion section.

## Discussion

This study determined FP and PN preferences amongst a selection of five PA assessment instruments. Preference for two instruments were identified; (1) GPPAQ and (2) 3Q. Reasons for preference were linked to a range of variables including; individual clinician PA status, knowledge/perceived competency in PA assessment, and features contained within each instrument.

Triangulation of data identified links between; (1) clinician PA status, (2) knowledge and perceived competency in PA assessment and (3) preference for PA instruments. Practice nurses maintained consistent preference for the GPPAQ instrument, had a lower proportion of personal PA (40 %) than FPs, and demonstrated limited knowledge/perceived competency and confidence in PA assessment. The reverse was the case for FPs and PNs who were recorded as physically active.

### Intrinsic variables

The relationship between the individual characteristics of clinicians and patient encounters is well documented [41–43]. Yet, little is known about the relationship between individual clinician characteristics and their impact on delivering PA assessment. This study identified a number of intrinsic demographic characteristics of clinicians that showed associations with instrument preferences including; clinician PA status and their level of competency regarding PA assessment.

Findings indicate that clinicians fit on a spectrum of high knowledge/competency through to limited knowledge/competency in terms of their ability to perform PA assessment. Clinicians categorised as physically inactive were associated with lower knowledge/competency of PA behaviour change, the reverse was the case for physically active clinicians. The GPPAQ demonstrated rudimentary support for clinicians, whereas the 3Q instrument suited those more familiar with the mechanisms of PA assessment. This study suggests that consideration of clinician knowledge/perceived competency and confidence of PA behaviour change could be addressed by simplifying terminology, and including relevant examples to guide the assessment process. A recent study conducted in the United Kingdom determined the usability of the GPPAQ amongst British GPs and nurses, specifically regarding its application within socio-economically disadvantaged populations [44]. Health professionals found the GPPAQ easy-to-use, particularly amongst patients with complex conditions who could benefit from PA behaviour change, however it suggested further enquiry around optimal methods of integration within routine practice [44].

For less knowledgeable clinicians, the time taken to complete an assessment is likely to be longer, particularly if the instrument does not support limited knowledge/competency. This finding is contrary to previous research [30, 45–47]. Whilst time is a limiting factor, this study suggests that it might be addressed if clinician knowledge and competency in PA assessment is augmented by the assessment instrument [30, 45–47].

### Extrinsic variables

There were differences observed between the GPPAQ and 3Q instrument variables (Table 3) determined in the preferred instrument analysis. These included theoretical orientation, terminology, number of questions, outcome measures, types of PA such as planned and/or incidental exercise, and inclusion of examples/scenarios to aid interpretation [24, 40]. Given the theoretical orientation of the GPPAQ lies within the context of family practice, it is not surprising there was a high preference for this instrument [40]. Interpretation of technical terminology, such as ‘vigorous’ and ‘moderate’ in the context of PA assessment proved difficult for some clinicians, specifically those linked with lower levels of individual PA

The patient population within each practice influenced clinician preferences, with clinicians ensuring the questions met the needs of current patients and/or encounters e.g. women with children, men and retired patients. Complex adaptive theory can be used to describe how clinicians considered the dynamic network of interacting agents presented during routine care such as balancing the need for acute care, with that of preventive care [48, 49]. This is compounded by variations in routine encounters according to patient demography e.g. patient gender, reason for visit or the complexity of conditions [50, 51].

The notion of a blanket approach to PA assessment, incorporating the entire patient population was not evident in this study. Consistent with complex adaptive practice, clinicians were selective, or decentralised in their approach, leveraging or drawing on a range of methods or situations to incorporate PA assessment into routine practice [52]. Examples of methods include clinicians initiating PA assessment following the delivery of poor pathology results or, during health assessments. Clinicians indicated that by using their preferred instrument, they would be able to integrate PA assessment into a given situation such as those outlined above. Theories of behaviour change and complexity for health promotion provide the best explanation for these findings, with clinicians using selective approaches to adapt to change by shifting one variable, such as the PA assessment instrument discussed here [4, 50, 51, 53].

### Strengths and limitations

Limitations of this study include the small sample size and potential generalizability beyond that of the geographical region in which the study was conducted. Despite this, there was equal representation of FPs and PNs. All clinicians had prior experience in referring patients for PA behaviour change via the local GPERS. As a result, it is recognised that this sample describes clinicians who may be more interested in PA assessment and preventative care, than the general population [29, 54, 55]. However, these health professionals are more likely to offer meaningful input regarding their application of PA assessment instruments, than those without prior involvement as they have an established commitment to preventative care. The geographical region where the study was conducted offers relative homogeneity with respect to other large, industrialised cities both in Australia and internationally. This extends beyond population profiles to rates of physical inactivity, rates of chronic disease and primary care systems [56–60].

Use of the TDF offers both strengths, and limitations. The strengths of this framework include the ability to draw on a range of relevant behaviour change and implementation research theories in one synthesised and accessible framework [39]. It is acknowledged that potential limitations may have impacted on the findings of this study; however the following efforts have been made to reduce any outliers [39]. The TDF was used as a structural framework for analysis only. Secondly, the investigators aimed to reduce associated limitations with data analysis by co-opting investigators skilled in behaviour change and implementation research skills.

## Conclusion

This study demonstrated preferences for two instruments, preferred for use in routine family-practice encounters. The GPPAQ was most preferred, followed by the 3Q for both FPs, and PNs. However, as experience in PA assessment increased both FPs and PNs reported increased satisfaction with the 3Q.

The GPPAQ has not previously been implemented in Australia, despite widespread application in the United Kingdom, whilst the 3Q has had an established position in Australian family practice through use in existing resources [24, 32].

Instrument preferences were influenced by a range of intrinsic and extrinsic variables. Intrinsic variables related to clinician knowledge/perceived competency of PA and/or individual PA levels. Extrinsic variables related to the content of instruments facilitating support for clinicians throughout the assessment process and limiting time taken to complete the assessment.

The outcomes of this study suggest that limited uptake of PA assessment in family practice may not be directly linked to clinician time restrictions, but associated with a range of intrinsic and extrinsic variables. It suggests that PA assessment may be related to variations in personal PA levels of clinicians, and that identification and integration of assessment instruments should be matched to their individual needs, acknowledging differences in physician knowledge/competency levels, and patient population.

Further research is required to quantify clinician knowledge of PA assessment to ensure instruments are appropriately graded to meet the needs for assessment.

## Appendix

**Table 4:** Time 1 and 2 interview schedule

Time 1: Interview schedule	Time 2: Interview schedule
Health professionals will be asked for their opinions about the following five instruments and their impression as a potential instrument for use by patients in their practice: 1. Active Australia. 2. Occupational Sitting and Physical Activity Questionnaire (OSPAQ). 3. 2-Question Physical Activity Questionnaire (2Q). 4. 3-Question Physical Activity Questionnaire (3Q). 5. General Practice Physical Activity Questionnaire (GPPAQ). A copy of each questionnaire provided to interviewee and given 10 minutes to review the content and format Investigator 1. From the selection of questionnaires provided, do you have any preferences on which ones you would rather use for patients in your practice? If so, why? 2. From the selection of questionnaires provided, are there any that you find difficult to understand? If yes, which ones and why? 3. From the selection of questionnaires provided, are there any specific questions that you would find difficult to explain to patients? If so, which ones and why? 4. Which of the 5 questionnaires would you feel most confident to use amongst patients in your practice and why? 5. Are there any questionnaires that you would be interested in finding out more about and possibly implement in routine practice? If so which ones and why? 6. If your most preferred questionnaire was provided to you by the method of your choice (e.g. Medical software), how often would you use this? 7. What sort of patients would you use this for?	1. How easy did you find the two questionnaires were to administer with patients? (Consider time taken and ease of use in completing the form) Questionnaire #1 Questionnaire #2 2. Did you have to explain, prompt or provide more information to patients about any of the questions? If so, which questionnaire and specific questions? 3. Did you have a preferred questionnaire and if so, which one and why? 4. How useful did you find the preferred questionnaire was in giving you an accurate picture of a patient’s current physical activity level 5. Would you use the preferred questionnaire again, if so what would you recommend to assist you in doing this?

## Abbreviations

2Q: 2-question physical activity questionnaire
3Q: 3-question physical activity questionnaire
AA: Active Australia
FP: Family physician
GP: General practitioner
GPERS: GP Exercise Referral Scheme
GPPAQ: General practice physical activity questionnaire
HREC: Human Research Ethics Committee
OSPAQ: Occupational sitting and physical activity questionnaire
PA: Physical activity
PCO: Primary care organization
PN: Practice nurse
TDF: Theoretical Domains Framework
